# Reciprocal Relationships between Sleep Problems and Problematic Smartphone Use in Taiwan: Cross-Lagged Panel Study

**DOI:** 10.3390/ijerph18147438

**Published:** 2021-07-12

**Authors:** Ji-Kang Chen, Wen-Chi Wu

**Affiliations:** 1 Department of Social Work, Chinese University of Hong Kong, Shatin, Hong Kong, China; jkchen@swk.cuhk.edu.hk; 2 Department of Health Promotion and Health Education, School of Education, National Taiwan Normal University, Taipei 106, Taiwan

**Keywords:** problematic smartphone use, sleep problems, adolescents, cross-lagged panel analysis

## Abstract

Prior studies have suggested a link between sleep problems and problematic smartphone use. However, the causal relationships between these two variables have not been identified, particularly in adolescence. Utilizing longitudinal panel data from Taiwan, this report examined the temporal relationships between sleep problems and problematic smartphone use among adolescents. One thousand and thirty-nine students (Grades 7–12) were surveyed at two-time points with a 6-month interval. The results of cross-lagged panel analysis showed that sleep problems at Time 1 significantly predicted problematic smartphone use at Time 2. Problematic smartphone use at Time 1 also significantly predicted sleep problems at Time 2. These findings applied to boys and girls and suggested that temporal relationships between sleep problems and problematic smartphone use among teenagers are reciprocal. Accordingly, increasing sleep quality may prevent future problematic smartphone use, while reducing problematic smartphone use may prevent sleep problems in adolescents.

## 1. Introduction

Adolescent sleep problems and problematic smartphone use pose major public concerns in Taiwan. A recent national survey in Taiwan indicated that approximately 54% of youth and adolescents reported having smartphone addiction or problematic smartphone use [1], and nearly one in five secondary school students experienced insomnia [2]. Sleep problems have been linked to the problematic use of electronic devices such as smartphones. An increasing number of studies have examined the relationships between sleep problems and problematic smartphone use [3]. Most of these studies have suggested that sleep problem is one of the negative outcomes of problematic smartphone use [4,5,6,7,8,9,10]. In contrast, other studies have argued that sleep problems contribute to problematic smartphone use [11,12]. To date, most studies on the relationships between sleep problems and problematic smartphone use have mainly utilized cross-sectional data [7,9,12,13]. Even though very few studies have used longitudinal data [14], these studies have failed to clarify the causal relationships between sleep problems and problematic smartphone use among teenagers. Therefore, whether sleep problems precede or result from problematic smartphone use is unclear. 

Several plausible explanations and studies have supported the link from problematic smartphone use to sleep problems [3,15]. For example, research has suggested that the blue monochromatic light emissions from the light-emitting diode (LED) of smartphones suppress the secretion of melatonin, which delays sleep onset. Experimental studies revealed that compared to the participants who read printed books before bedtime, participants who had been exposed to blue light from the LED screen of smartphones showed delayed melatonin secretion and decreased sleepiness [16,17]. In addition, problematic smartphone use before bedtime can induce sleep procrastination. Some studies have found that using screens before bedtime may phase-shift the circadian clock and increase alertness [11,17,18]. Similarly, surfing web pages, watching videos, or playing games on smartphones can contribute to pre-sleep arousals among adolescents. Researchers have revealed that adolescents with pre-sleep arousals elicited by excessive screen use had a poorer sleep quality and more symptoms of insomnia [19,20]. Finally, problematic smartphone use may replace activities promoting good sleep quality (e.g., sports and exercises). Cross-sectional studies have indicated a positive association of problematic smartphone use with physical inactivity and sedentary behavior [20,21]. Experimental research has also revealed that exercise intervention decreased problematic smartphone use [22]. 

On the other hand, sleep problems may increase the likelihood of problematic smartphone use. Although relatively less research has investigated sleep problems as an antecedent of problematic smartphone use, researchers have proposed several theories. For example, circadian preference might affect problematic smartphone use. Randler et al. [11] found that adolescents who are evening types (go to bed late and wake up late) were more likely to use smartphones problematically compared to those who are morning types (getting up early in the morning and getting tired earlier in the evening). In addition, individuals may use smartphones when they have difficulties falling asleep. Tavernier and Willoughby [23] observed that university students with sleep problems spent more time on social networking sites using their mobile devices to cope with their sleep problems. 

To the best of our knowledge, only two relevant academic reports have investigated the bi-directional associations of sleep problems with Internet addiction and social media use utilizing longitudinal data [23,24]. However, the findings of these two studies yielded contradictory results. One study examining the long-term association between sleep problems and Internet addiction among 1253 children in grades 3 to 8 revealed a bi-directional relationship [24]. However, the other study on the associations between sleep problems and social media use among 942 university students showed that sleep problems predicted longer time spent on social media, but not vice versa [23]. Important gaps remain in the research on the causal link between sleep problems and problematic smartphone use among adolescents. Understanding the temporal relationship between sleep problems and problematic smartphone use among adolescents can inform future health promotion programs for adolescents.

This study examined the prospective causal relationships between sleep problems and adolescents’ problematic smartphone use in Taiwan using longitudinal panel data. Additionally, this study examined whether the temporal relationships between sleep problems and problematic smartphone use varied by gender. Evidence from cross-sectional studies on the association between sleep problems and problematic smartphone use among adults is mixed. For example, Chang and Choi [9] reported that problematic smartphone use is associated with deprived sleep quality among male but not female participants [9]; however, Chen, Liu, Ding, Ying, Wang, and Wen found a significant association between deprived sleep quality and smartphone addiction in male and female medical college students [12]. No previous study has examined whether the temporal relationships between sleep problems and problematic smartphone use vary between male and female adolescents using longitudinal data. This work utilized cross-group comparison to evaluate gender differences in the temporal relationships between sleep problems and problematic smartphone use. 

## 2. Material and Methods

### 2.1. Participants

The secondary data in this study were part of a longitudinal project conducted in northern Taiwan. The project followed 7th graders in junior high schools and 10th graders in senior high schools for four semesters from October 2019 to June 2021. A stratified clustered random sampling procedure was conducted to select the participating schools. The junior high and senior high schools in Taoyuan City and New Taipei City were ranked according to the school size. Thirteen junior high schools and four senior high schools in Taoyuan City and 12 junior high schools and five senior high schools in New Taipei City were randomly selected. In total, 34 out of 169 schools were selected. Next, two classes were randomly sampled in each school, except for a school with only one 7th grade class. All students in the selected classes were included. Overall, 67 classes comprising 1712 students participated in the first and second wave of the project, while 350 students declined to participate in the third wave. The reasons for declining to participate in the third wave included no time, no interest, and sick leave.

The project adopted an online survey procedure to obtain the students’ information. Surveycake.com was used to build an online fill-in questionnaire. The trained instructors wrote the link to this online questionnaire on the whiteboard in the computer classroom of each school, and students typed this link to the browser. After they entered the webpage of the questionnaire, they saw a consent form on the first page. They were required to read the content and click the icon if they agreed to participate. If they declined to join the project, they could surf the Internet quietly without affecting the other students. The whole procedure was conducted by standardized trained instructors to avoid the influence of the teachers or other school staff. The respondents were encouraged to respond honestly. 

The Research Ethics Office of National Taiwan Normal University (No. 201906HS007) reviewed and approved the procedures, informed consent forms, questionnaire, and other ethical concerns.

The study analyzed 1039 junior-high and senior-high school students who completed the second-and third-wave survey because the sleep problems were only measured at these two waves. Among the 1039 participants, 492 (47.4%) students were boys, and 547 (52.6%) were girls.

### 2.2. Measures

#### 2.2.1. Sleep Problems

The measure of sleep problems was adopted from the Insomnia Severity Index-Chinese version (ISI-C) [25,26]. ISI-C measures the respondents’ self-perceived symptoms and outcomes of insomnia experienced “in the last two weeks.” Seven items including (a) the severity of sleep-onset (initial); (b) sleep maintenance (middle); (c) early morning awakening (terminal) problems; (d) satisfaction with current sleep pattern; (e) interference with daily functioning; (f) noticeability of impairment attributed to the sleep problem; and (g) level of distress caused by the sleep problem. Each item was scored on a 0 to 4 scale. The total score ranged from 0 to 28. A higher score indicated a higher level of insomnia. The Cronbach’s alpha was satisfactory (α = 0.78 at Time 1 and α = 0.81 at Time 2). 

#### 2.2.2. Problematic Smartphone Use 

This scale was adopted from the Smartphone Addiction Scale short version (SAS-SV) consisting of 10 items measured on a six-point Likert scale (1: “strongly disagree” and 6: “strongly agree”) [27]. This scale assesses daily-life disturbance, withdrawal, overuse, and tolerance of using a smartphone. Sample items include “Missing planned work due to smartphone use”, “Having a hard time concentrating in class while doing assignments or while working due to smartphone use”, and “Having my smartphone in my mind even when I am not using it”. The Cronbach’s alpha was satisfactory (α = 0.86 at Time 1 and α = 0.87 at Time 2).

#### 2.2.3. Control Variables

Mother’s and father’s education level were categorized as 1 = elementary school or below, 2 = junior high school, 3 = senior high school, 4 = associate degree, 5 = bachelor’s degree, and 6 = master’s degree or above. Father’s and mother’s job status included having no job (coded 1), having a part-time job (coded 2), and having a full-time job (coded 3). School type included junior high (coded 1) and senior high school (coded 2). 

### 2.3. Analysis Plan

First, IBM Statistical Package for the Social Science (SPSS) version 25.0 [28] was used to analyze the descriptive analysis (e.g., frequency, percentage, mean, standard deviation, and range) and inter-correlations between variables in this study. Next, a cross-lagged panel analysis [29,30] was conducted using structural equation modeling (SEM) in AMOS 25 [31] to assess the reciprocal associations between sleep problems and problematic smartphone use. In the cross-lagged panel analysis, sleep problems and problematic smartphone use at Time 2 were regressed onto the same variable at Time 1. In addition, relationships of sleep problems with problematic smartphone use were analyzed using a six-month lag time while controlling for opposite relationships. This cross-lagged panel analysis was chosen because it allowed us to evaluate the temporal relationships between sleep problems and problematic smartphone use. We also included school types, parents’ educational levels, and job status as control variables in the cross-lagged model.

A cross-group SEM analysis was further conducted to investigate gender differences in the association between sleep problems and problematic smartphone use in the cross-lagged model. The full information maximum likelihood procedure was utilized to estimate missing values [31]. In SEM, the *χ*2 statistic is generally employed to evaluate the goodness of the model. However, it is highly sensitive to the sample size. Previous studies have suggested that other goodness-of-fit indices such as the comparative fit index (CFI) [32], normed-fit index (NFI) [33], incremental fit index (IFI) [34], and the root mean square error of approximation (RMSEA) [35] are more appropriate to evaluate the model fit. Fit values of CFI, NFI, and IFI greater than 0.95 and RMSEA smaller than 0.06 are considered acceptable, indicating a good model fit [36].

## 3. Result

Descriptive statistics for each study variable and bivariate correlations between variables are shown in Table 1 and Table 2, respectively. The sex distribution of the participants was almost equivalent (52.6% for boys and 47.4% for girls). About eighty percent of participants were junior high school students. Of the parents, most of them graduated from senior high schools (38.1% for mothers and 38.8% for fathers) and 75% of fathers and 61.3% of mothers had full-time jobs. The cross-sectional correlations between sleep problems and problematic smartphone use were positive. The bivariate correlations between sleep problems and problematic smartphone use also was also shown to be positive across time.

### 3.1. Reciprocal Relations between Sleep Problems and Problematic Smartphone Use

**Figure 1:**
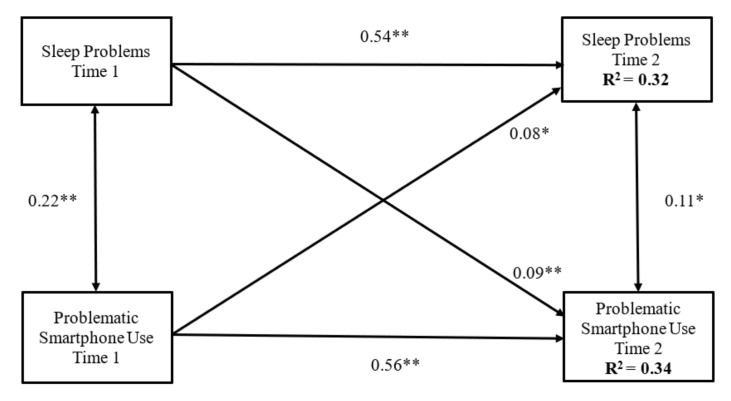
The cross-lagged analyses between sleep problems and problematic smartphone use. The model controlled for school types, parents’ education level, and parents’ job status. **p < 0.001. *p < 0.01.

A cross-lagged panel model was conducted to examine the reciprocal relationships between sleep problems and problematic smartphone use among adolescents, controlling for school types as well as fathers and mothers’ educational background and job status. The model, shown in Figure 1, revealed a good fit to the data, χ2 (10) = 35.96, *p* < 0.01, CFI = 0.98, NFI = 0.97, IFI = 0.98, and RMSEA = 0.05. 

Figure 1 demonstrates that all autoregressive effects were significant (from sleep problems at Time 1 to Time 2, β = 0.54, *p* < 0.001; from problematic smartphone use at Time 1 to Time 2, β = 0.56, *p* < 0.001), indicating that sleep problems and problematic smartphone use were relatively stable over time. In addition, sleep problems at Time 1 predicted adolescents’ problematic smartphone use at Time 2 (β = 0.09, *p* < 0.01) and problematic smartphone use at Time 1 also significantly predicted sleep problems at Time 2 (β = 0.08, *p* < 0.01), suggesting the reciprocal relations between sleep problems and problematic smartphone use. The model accounted for 32% and 34% of the variance in sleep problems and problematic smartphone use. Within-time or synchronous correlations between sleep problems and problematic smartphone use at Time 1 were significant (β = 0.22, *p* < 0.001). The within-time correlation between sleep problems and problematic smartphone use at Time 2 was smaller but still significant (β = 0.11, *p* < 0.01). Among the covariates not shown in Figure 1, fathers’ education level at Time 1 correlated negatively with problematic smartphone use at Time 1 (β = −0.11, *p* < 0.01). Mothers’ job status at Time 1 correlated negatively with problematic smartphone use at Time 1 (β = −0.09, *p* < 0.01). School type at Time 1 correlated positively with sleep problems (β = 0.12, *p* < 0.001) and problematic smartphone use (β = 0.10, *p* < 0.001) at Time 1, respectively. All other covariates were not significantly associated with the primary variables (β ranges from −0.06 to 0.03, and all *p* values were smaller than 0.05).

### 3.2. Boys vs. Girls

**Figure 2:**
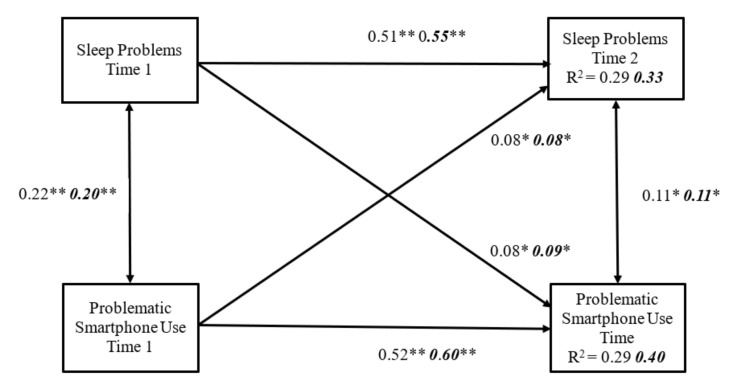
The cross-lagged model with bidirectional effects between sleep problems and problematic smartphone use between sexes. The model controlled for school types, parents’ education level, and parents’ job status. The coefficients in regular print and those in bold italics, represent, respectively, the results for the male and the female samples. ** *p* < 0.001, * *p* < 0.01.

A comparative analysis was conducted to investigate whether the cross-lagged model and cross-lagged relations between sleep problems and problematic smartphone use varied between boys and girls. In this analysis, all structural paths between Time 1 and Time 2 in the cross-lagged model were constrained (i.e., the path from sleep problems at Time 1 to problematic smartphone use at Time 2 and the path from problematic smartphone use at Time 1 to sleep problems at Time 2) simultaneously for boys and girls. The model demonstrated good fit to the data: χ2 (22) = 47.280, *p* < 0.01, CFI = 0.98, NFI = 0.97, IFI = 0.98, and RMSEA= 0.033. Next, we released these two constrained paths together, and the results of model fit were: χ2 (20) = 42.766, *p* < 0.01, CFI = 0.98, NFI = 0.97, IFI= 0.98, and RMSEA = 0.033. The differences in chi-square values between unconstrained and constrained models were non-significant [Δχ2 (2) = 4.51, *p* > 0.05], suggesting no significant differences in the cross-lagged model between boys and girls. Figure 2 shows the results for the constrained model, revealing that the regression coefficients for each path and explained variances on sleep problems and problematic smartphone use were similar for boys and girls.

## 4. Discussion

Although an increasing number of studies have examined the link between sleep problems and problematic smartphone use, no studies have investigated the causal relationships between these two variables among adolescents over time. As a result, whether the sleep problem is the antecedent or the adverse effect of adolescents’ problematic smartphone use remains unanswered. Utilizing two-wave longitudinal panel data from Taiwan, this study conducted a cross-lagged panel analysis to examine the temporal relationships between sleep problems and problematic smartphone use among adolescents. Overall, this study showed evidence to support that adolescents’ sleep problems lead to problematic smartphone use and vice versa. That is, the temporal relationships between sleep problems and problematic smartphone use among adolescents are reciprocal. The findings were also relevant for boys and girls.

### 4.1. Reciprocal Relationships between Problematic Smartphone and Sleep Problems

Consistent with previous cross-sectional studies [4,5,11,12], this study confirmed the concurrent correlations between problematic smartphone use and sleep problems. Moreover, this study established the path from problematic smartphone use to sleep problems, echoing the conclusions of the previous studies, which proposed the blue-ray effect, circadian clock shifting effect, pre-sleep arousal effect, and sleep-promoting-activity displacement effect to explain the above relationships [3,15]. This study further proved the link from sleep problems to problematic smartphone use, consistent with the proposition that adolescents may use smartphones when they have difficulties falling asleep [37]. Combining these findings, our results suggest that problematic smartphone use acts as an antecedent of sleep problems and vice versa. 

### 4.2. Gender Differences

Our results revealed that gender does not moderate the cross-lagged association between sleep problems and adolescents’ problematic smartphone use. The findings are consistent with previous cross-sectional studies indicating that the association between sleep quality and smartphone use is similar across genders [12]. The findings further confirm the temporal relationships between sleep problems and problematic smartphone use for boys and girls. 

### 4.3. Limitations

This study needs to be interpreted in light of some limitations. First, the two-wave panel data in this study were collected at 6-month intervals. Future work may consider longitudinal panel studies with three and more waves or employ longer follow-up periods to confirm the bi-directional associations of sleep problems with problematic smartphone use among adolescents. Second, previous studies have commonly utilized student reports to measure sleep problems and problematic smartphone use. It is debatable whether such measures grasp the true essence of these two constructs [3]. Future research may measure adolescent sleep problems and problematic smartphone use by collecting data from different sources such as the parents to increase the validity of the design [3]. Third, although our study indicated the reciprocal relationships between adolescent sleep problems and problematic smartphone use over time, future research needs to examine the potential psychosocial mechanisms underlying the link between sleep problems and problematic smartphone use. Fourth, sleep problems were measured subjectively while sleep duration and other sleep patterns (e.g., irregular sleep-wake schedules and sleep onset latency) were not included in this study. Future researchers may consider examining the associations between these variables with problematic smartphone use among adolescents [14]. Fifth, we did not control for variables that may be correlated with sleep problems and problematic smartphone use such as psychological distress and academic stress [13]. Future studies may also control for these factors in the model. Sixth, we did not collect information about smartphone types and screen size in this longitudinal survey. Future researchers may consider collecting such information and controlling them in the model. Finally, the reciprocal relationships between sleep problems and problematic smartphone use found in this study were based on data collected from Taiwanese teenagers. Researchers may replicate our findings in different countries or cultures to enhance their generalizability. 

## 5. Conclusions

Current findings confirmed the bi-directional associations between sleep problems and problematic smartphone use after controlling for their concurrent association. In addition, the same reciprocal associations were found for both genders. Adolescents with problematic use of smartphones may experience sleep problems for several reasons (e.g., the effect of blue-rays and pre-sleep arousal. Those who suffer from sleep problems may resort to using smartphones whenever they are having a hard time falling asleep, leading to problematic smartphone use. The findings have important implications for developing prevention and intervention programs targeting sleep problems and problematic smartphone use among adolescents. Health care professionals and educators need to pay attention to the reciprocal causal relationships between sleep problems and problematic smartphone use and seek remedy to reduce at least one of them, since solving one may reduce the other [38,39]. 

## Figures and Tables

**Table 1:** Descriptive statistics of study variables (*n* = 1039).

Variable	*N* (%)	*Mean* (SD)	Range
W1 Sleep problem		5.36 (4.29)	0–25
W2 Sleep problem		5.11 (4.32)	0–25
W1 Problematic smartphone use		31.23 (10.55)	10–60
W2 Problematic smartphone use		31.36 (10.60)	10–60
W1 Sex			
Boys	492 (47.4%)		
Girls	547 (52.6%)		
W1 School type			
Junior high school	822 (79.1%)		
Senior high school	217 (20.9%)		
W1 Fathers’ education level			
Elementary school	18 (1.7%)		
Junior high school	104 (10.0%)		
Senior high	396 (38.1%)		
Associate degree	114 (11.0%)		
Bachelor degree	202 (19.4%)		
Master degree or above	84 (8.1%)		
Don’t know (Missing)	121 (11.6%)		
W1 Mothers’ education level			
Elementary school	24(2.3%)		
Junior high school	94(9.0%)		
Senior high school	403 (38.8%)		
Associate degree	161 (15.5%)		
Bachelor degree	197 (19.0%)		
Master degree or above	54 (5.2%)		
Don’t know (Missing)	106 (10.2%)		
W1 Fathers’ job status			
No job	65 (6.3%)		
Part-time	61 (5.9%)		
Full-time	779 (75.0%)		
Don’t know (Missing)	134 (12.9%)		
W1 Mothers’ job status			
No job	177 (17.0%)		
Part-time	106 (10.2%)		
Full-time	637 (61.3%)		
Don’t know (Missing)	119 (11.5%)		

Note. Standard deviations are in parentheses.

**Table 2:** Correlations among all study variables.

Variables.	1	2	3	4	5	6	7	8	9	10
1. W1 Sleep problem	-	0.54 **	0.23 **	0.22 **	0.13 **	0.12 **	–0.04	–0.00	0.01	–0.05
2. W2 Sleep problem		-	0.21 **	0.24 **	0.10 **	0.11 **	0.02	0.05	–0.03	–0.07 *
3. W1 Problematic smartphone use			-	0.58 **	0.10 **	0.11 **	–0.12 **	–0.09 **	0.00	–0.09 **
4. W2 Problematic smartphone use				-	0.07 *	0.17 **	–0.13 **	–0.13 **	–0.02	–0.05
5. W1 Sex					-	–0.03	–0.06	–0.03	–0.00	–0.06
6. W1 School type						-	–0.06	–0.04	0.02	0.03
7. W1 Fathers’ education level							-	0.60 **	0.13 **	0.05
8. W1 Mothers’ education level								-	0.12 **	0.15 **
9. W1 Fathers’ job status									-	0.04
10. W1 Mothers’ job status										-

** *p* < 0.01, * *p* < 0.05

## Data Availability

The data presented in this study are available on request from the corresponding author and with the permission of the Research Ethics Committee, National Taiwan Normal University.
